# Comparative analysis of the root and leaf transcriptomes in *Chelidonium majus* L.

**DOI:** 10.1371/journal.pone.0215165

**Published:** 2019-04-15

**Authors:** Helen Pourmazaheri, Aboozar Soorni, Bahram Baghban Kohnerouz, Nafiseh Khosravi Dehaghi, Enayatollah Kalantar, Mansoor Omidi, Mohammad Reza Naghavi

**Affiliations:** 1 Department of Plant Breeding and Biotechnology, College of Agriculture, University of Tabriz, Tabriz, Islamic Republic of Iran; 2 Department of Pharmacognosy, Faculty of Pharmacy, Alborz University of Medical Sciences, Karaj, Islamic Republic of Iran; 3 Department of Biotechnology, College of Agriculture, Isfahan University of Technology, Isfahan, Iran; 4 Department of Microbiology and Immunology, Faculty of Medicine, Alborz University of Medical Science, Karaj, Islamic Republic of Iran; 5 Agronomy and Plant Breeding Department, Agricultural & Natural Resources College, University of Tehran, Karaj, Islamic Republic of Iran; ICAR - National Research Center on Plant Biotechnology, INDIA

## Abstract

*Chelidonium majus* is a traditional medicinal plant, which commonly known as a rich resource for the major benzylisoquinoline alkaloids (BIAs), including morphine, sanguinarine, and berberine. To understand the biosynthesis of *C*. *majus* BIAs, we performed *de novo* transcriptome sequencing of its leaf and root tissues using Illumina technology. Following comprehensive evaluation of *de novo* transcriptome assemblies produced with five programs including Trinity, Bridger, BinPacker, IDBA-tran, and Velvet/Oases using a series of k-mer sizes (from 25 to 91), BinPacker was found to produce the best assembly using a k-mer of 25. This study reports the results of differential gene expression (DGE), functional annotation, gene ontology (GO) analysis, classification of transcription factor (TF)s, and SSR and miRNA discovery. Our DGE analysis identified 6,028 transcripts that were up-regulated in the leaf, and 4,722 transcripts that were up-regulated in the root. Further investigations showed that most of the genes involved in the BIA biosynthetic pathway are significantly expressed in the root compared to the leaf. GO analysis showed that the predominant GO domain is “cellular component”, while TF analysis found bHLH to be the most highly represented TF family. Our study further identified 10 SSRs, out of a total of 39,841, that showed linkage to five unigenes encoding enzymes in the BIA pathway, and 10 conserved miRNAs that were previously not detected in this plant. The comprehensive transcriptome information presented herein provides a foundation for further explorations on study of the molecular mechanisms of BIA synthesis in *C*. *majus*.

## Introduction

*Chelidonium majus* L. is an herbaceous medicinal plant belonging to the botanical family Papaveraceae. *C*. *majus* is widely distributed in Europe and Western Asia and also as an introduced species in Northern America. The species is commonly known as celandine, greater celandine, celandine poppy, elon-wort, felon-wort, rock poppy, swallow-wort, and tetter-wort. *C*. *majus* is highly toxic due to the presence of various secondary metabolites in the roots and stems, but is used in both traditional and modern medicines [1].

Pharmacological properties ascribed to *C*. *majus* include anti-viral [2], anti-bacterial [3], anti-fungal [4], anti-protozoal and radioprotective [5], anti-inflammatory [6], anti-alzheimer [7], anti-cancer [8], hepatoprotective [9], and natriuretic and antidiuretic effects [10]. The diverse array of secondary metabolites present in *C*. *majus* is responsible for its therapeutic properties. Alkaloids are the most common group of secondary metabolites present in *C*. *majus*. Chelidonine, berberine, sanguinarine, coptisine, chelerythrine, and protopine, are among the various alkaloids synthesized by *C*. *majus* [11]. Flavonoids, saponins, vitamins (e.g. vitamin A and C), mineral elements, sterols, and acids and their derivatives [12] are other secondary metabolites present in *C*. *major* as well.

Transcriptome sequencing can be effectively utilized to identify and characterize pathways associated with the biosynthesis of secondary metabolites in plants [13–15], and enables the exploration of gene sequence and expression levels in an organism that lacks genomic resources [16–17]. A *de novo* transcriptome assembly, coupled with a liquid chromatography–electrospray ionization-tandem mass spectrometry (LC–ESI-MS/MS) proteomic approach, has been previously performed for *C*. *majus* to examine its protein composition, which showed novel defense-related proteins characteristic of its latex [18]. Also, Hagel et al. (2015) established an essential resource for the elucidation of benzylisoquinoline alkaloids (BIA) metabolism from the transcriptomes of 20 BIA-accumulating plants, but the structural diversity of the alkaloids and their biosynthetic pathways are not well studied in *C*. *majus*.

Considering the benefits of RNA sequencing technology, we used the root and leave tissues as the basic materials to generate RNA-seq reads using Illumina HiSeq 2000 to obtain a better understanding about genes involved in the BIA biosynthesis pathway We also mined the assembly to identify expressed sequence tag simple sequence repeats (EST-SSRs) and miRNAs that have not yet been characterized in *C*. *majus*.

## Materials and methods

### Plant materials, RNA extraction, and nucleotide sequencing

The *Chelidonium majus* tissues used in this study were collected from a high producer of chelidonine (Voucher number: IBRCP1006619), Mahmudabad-Amol, Mazandaran, Iran (Longitude coordinates: 52 17' 0.9", Latitude coordinates: 36 35' 15.1 ") [19]. To collect samples, plants were grown in the greenhouse facilities (28°C day/20°C night under natural light conditions) of the Iranian Biological Resources Center (IBRC) in Alborz, Iran. The root and leaf tissues were harvested from the plants, washed thoroughly with sterile water, frozen in liquid nitrogen and immediately stored at -80°C. Total RNA from the harvested plant materials was extracted using TRIzol^®^ Reagent according to the manufacturer’s instructions (Invitrogen, USA). RNA samples were sent to the Beijing Genomic Institute (BGI) for transcriptome sequencing. Libraries were constructed using illumina TruSeq RNA sample preparation kit, while sequencing was performed with the Illumina HiSeq 2000 platform to generate paired-end (2×150 base) reads.

### *De Novo* transcriptome assembly

We obtained a draft transcriptome from the raw RNA sequencing data using five popular assembly programs including (1) Trinity v. 2.4.0 [16], (2) Velvet v.1.2.10 and Oases v.0.2.09 [20–21], (3) IDBA-tran [22], (4) Bridger [23], and (5) BinPacker [24]. Trinity was used with a fixed k-mer size of 25 as suggested by the authors. Oases-Velvet and IDBA-tran were used with a series of k-mer sizes from 25 to 91 and with an increment of 2. For BinPacker and Bridger we used two k-mer sizes (25 and 27). All tools were run with default settings and only assembled transcripts longer than 200 bp were retained. Subsequently, the most basic metrics for transcriptome assemblies including contig number, length distribution, assembly size, percentage of reads that could be mapped back to the transcriptome assembly (RMBT), and N50 were assessed and compared for all assemblies.

### Gene expression levels and transcript annotation

The RNA-seq by Expectation Maximization (RSEM) package was used to estimate gene expression levels based on the mapping of RNA-seq reads to the assembled transcriptome [25]. To estimate the individual transcript abundances, the RNA-seq reads had first to be aligned to the transcriptome assembly. After indexing the reference transcriptome, separately, fastq files from the individual libraries of each sample were mapped to the final transcript set using script align_and_estimate_abundance.pl. The program Bowtie was used to generate alignments for each sample. Combining the read counts from all samples into a matrix was performed using script abundance_estimates_to_matrix.pl [16, 25]. Finally, identification of differentially expressed genes was carried out using run_DE_analysis.pl, which involves the Bioconductor package EdgeR in the R statistical environment [26–27]. Transcripts with very low read counts were filtered out across all libraries. Gene expression values were measured in FPKM (fragments per kilobase of transcript per million reads mapped) [26,28] and were used to make pairwise comparisons. Clustering analysis was performed on the differentially expressed genes, with FDR and the logFC cutoff defined by the–P 1e-3 -C 2 parameters using analyze_diff_expr.pl script.

Functional annotation of the *de novo* transcriptome was conducted using TransDecoder v2.0.1 to predict open reading frames (ORFs) at least 100 amino acids long, and the Trinotate pipeline v3.0.2 (http://trinotate.github.io/) was used to annotate the predicted ORFs using the following programs: BLASTX v2.2.29 and BLASTP v2.2.29 to search against Swissprot-Uniprot database [29], Hmmer v.3.1b2 to identify protein domains (PFAM) [30–31], SignalP v.4.1 to predict the presence of signal peptides [32], Tmhmm v.2.0c for prediction of transmembrane helices in proteins [33], and Rnammer v.1.2 to predict ribosomal RNA [30]. All results from the bioinformatics analyses performed above were imported into a Trinotate SQLite database. To obtain Gene Ontology (GO) annotations, we used the Trinotate-integrated UniProtKB GO annotations and WEGO software [34] for GO functional classification.

### TF identification and EST-SSR analysis

Homology searches against PlantTFDB using BLASTx with a cut-off *E-value* of 1e−5 were performed in order to identify transcription factors [35]. The assembled sequences were scanned to identify single sequence repeats (EST-SSRs) using the MIcroSAtellite Identification Tool (MISA, http://pgrc.inpk-gatersleben.de/misa/) [36]. For this purpose, a FASTA file containing all of the assembled sequences was used as the input file in MISA Perl script to screen for EST-SSRs with motifs of 1 to 6 nucleotides and a minimum repeat number of 10, 6, 5, 5, 5, and 5, respectively. PCR primers were designed using Primer3 [37]. The parameters for designing primers were as follows: PCR product size range of 100 to 300 bp; primer length of 18–25 nucleotides; annealing temperature between 55 and 62°C with 57°C as the optimum melting temperature.

### *In silico* miRNA identification

To identify potential miRNAs in *C*. *majus*, transcripts and previously-known plant miRNAs from the miRBase database [38] were initially clustered using CD-HIT-EST [39] with the following parameters: c = 1, n = 10, d = 0, and M = 16000. The clustered sequences were then aligned against non-redundant miRNAs using BLASTn v 2.2.30 [29]. The obtained hits with alignment length > = 20, e-value threshold ≤ 0.001, and without mismatches and gaps were considered for extracting the precursor sequences (pre-miRNA). A sliding window of about 400 nt from the region 200 nt upstream of the beginning of the mature miRNA to 200 nt downstream of the miRNA from the filtered sequences was then used as a query in BLASTX searches against the NCBI non-redundant protein database to remove protein coding sequences. The secondary structures of the retained sequences were predicted using the web server mfold (Zuker, 2003). Only sequences with the following criteria were considered as potential miRNA precursors: (1) > = 20 nt mature miRNA sequence within one arm of the hairpin (2) with higher negative minimal free energies and higher MFEIs [40], (3) no more than six mismatches with the opposite miRNA, and (4) no loop or break in miRNA sequences. In the last step, we used the web tool psRNA-target (http://bioinfo3.noble.org/psRNATarget/) to predict the potential miRNA targets.

### Orthogroup identification

We used OrthoFinder [41] with the default parameters, aligned sequences with MAFFT v 7.271 [42] and built trees with FastTreeMP v 2.1.8 [43], to identify conserved orthogroups for eight species, including *Argemone mexicana*, *Papaver bracteatum*, *Eschscholzia californica*, *Glaucium flavum*, *Stylophorum diphyllum*, *Sanguinaria canadensis*, and *Corydalis cheilanthifolia* published previously along with *C*. *majus*. The corresponding transcriptome assemblies for seven species were downloaded from www.phytometasyn.ca [44]. The predicted protein sequences were obtained using TransDecoder v2.0.1 (http://transdecoder.sourceforge.net/). The rooted species tree was drawn using Dendroscope v 3.5.9 [45].

## Results and discussion

### Short-read sequencing and *de novo* transcriptome assembly

A total of 188.98 million clean PE RNA-seq reads of 150 bp in length with quality scores of >Q20 were obtained after sequencing root and leaf tissues on Illumina HiSeq 2000^™^ platform. Subsequently all 188.98 million of the high quality were used for *de novo* assembly using different packages.

The primary assembly statistics showed variable patterns of performance with the different tools; for example, Trinity produced the largest number of contigs with the highest number of bps, followed closely by Bridger (Table 1). The number of predicted transcripts is strongly affected by the k-mer size [46]. With Velvet/Oases, the number of predicted transcripts dropped from 325,276 with k-mer 25 to 94,116 with k-mer 91, similarly to previously reported results [46–48]. However, using IDBA-tran, the number of contigs generally increased with increasing k-mer size (168,305 contigs with k-mer 25, and 210,145 with k-mer 91). Some previous studies have indicated that N50, a metric commonly used in genome assembly, is not suitable for transcriptome assembly, because longer N50 values may indicate a high level of chimerism [6,49], although it has also been observed that larger N50s can reflect a higher quality assembly [50–51]. BinPacker gave the largest N50 compared to Trinity and Bridger. With increased k-mer size, the N50 increased for all Velvet/Oases and IDBA-tran k-mer assemblies. The total assembly length showed a similar trend N50 for IDBA-tran, while for Velvet/Oases with increasing k-mer size, the number of bps increased up to k = 45, at which point the number of bps declined. Across all assembly strategies performed using the different programs, Trinity, Bridger, and BinPacker consistently produced similar percentages of paired-end reads that mapped back to the relative assembly, ranging from 90.22 to 93.78%. Assemblies produced by Velvet/Oases had the lowest percentage of mapped reads (>70%). BinPacker was faster, compared to Trinity and Bridger. These conflicting patterns show that the outputs of the assembly programs can be quite variable.

**Table 1:** Statistical summary of *de novo* transcriptome assemblies for three assembly programs.

Tools	Trinity	Bridger		BinPacker	
Kmer Size	25	25	27	25	27
Number of contigs	392,555	336,021	334,664	232,701	227,138
Total size (Mb)	220.67	207.94	204.34	216.24	208
Maximum length (bp)	16,238	37,322	25,856	35,898	34,163
Minimum length (bp)	224	201	201	200	200
Average length (bp)	562.15	618.84	610.58	929.27	915.75
N50 length (bp)	674	1,013	993	1,585	1,552

Based on the assembly statistics, the assembly generated by BinPacker with k-mer 25, which had the highest N50 value (1,585 bp), average transcript length, and RMBT percentage, whilst keeping fewer number of contigs (232,701) and larger total assembly size (216.24 Mbp) as long as possible was selected for downstream analysis.

### Identification of differentially expressed genes (DEGs)

The identification of DEGs was performed by estimating individual transcript abundance by mapping the cleaned reads back to the assembled transcripts with RSEM, and their expression levels were represented as FPKM values. More than 93% of trimmed reads in the four libraries could be mapped to the transcriptome assembly successfully, which indicates the quality of the *de novo* transcriptome assembly. Digital abundance analysis identified 10,750 unique transcripts as being significantly different between leaves and roots with two biological replicates where the criteria for FDR was set to 0.001 and fold-change was set to 2^(2) or 4-fold; 6,028 transcripts were up-regulated in leaf and 4,722 transcripts in root. The fold-change ranged from 2 to 14.

In order to identify the active pathways represented in the leaf and root transcriptome of *C*. *majus*, the DEG sequences were used as queries in searches against the Kyoto Encyclopedia of Genes and Genomes (KEGG) pathway database. A total of 3,379 transcripts (31.43%), 1,828 transcripts being up-regulated in the leaf and 1,507 transcripts up-regulated in the root, were assigned to 354 pathways. These canonical pathways were classified into six categories (Metabolism, Genetic Information Processing, Environmental Information Processing, Cellular Processes, Organismal Systems, and Human Diseases) and 43 sub-categories. Among the pathways, “metabolic pathways” (with 347 transcripts), “biosynthesis of secondary metabolites” (192 transcripts), and “ribosome” (101 transcripts) were the most abundant. Since *C*. *majus* produces a major group of secondary metabolites (especially alkaloids), it was necessary to identify the most active genes involved in the metabolic pathways.

### Genes related to alkaloid biosynthesis pathways

**Fig 1:**
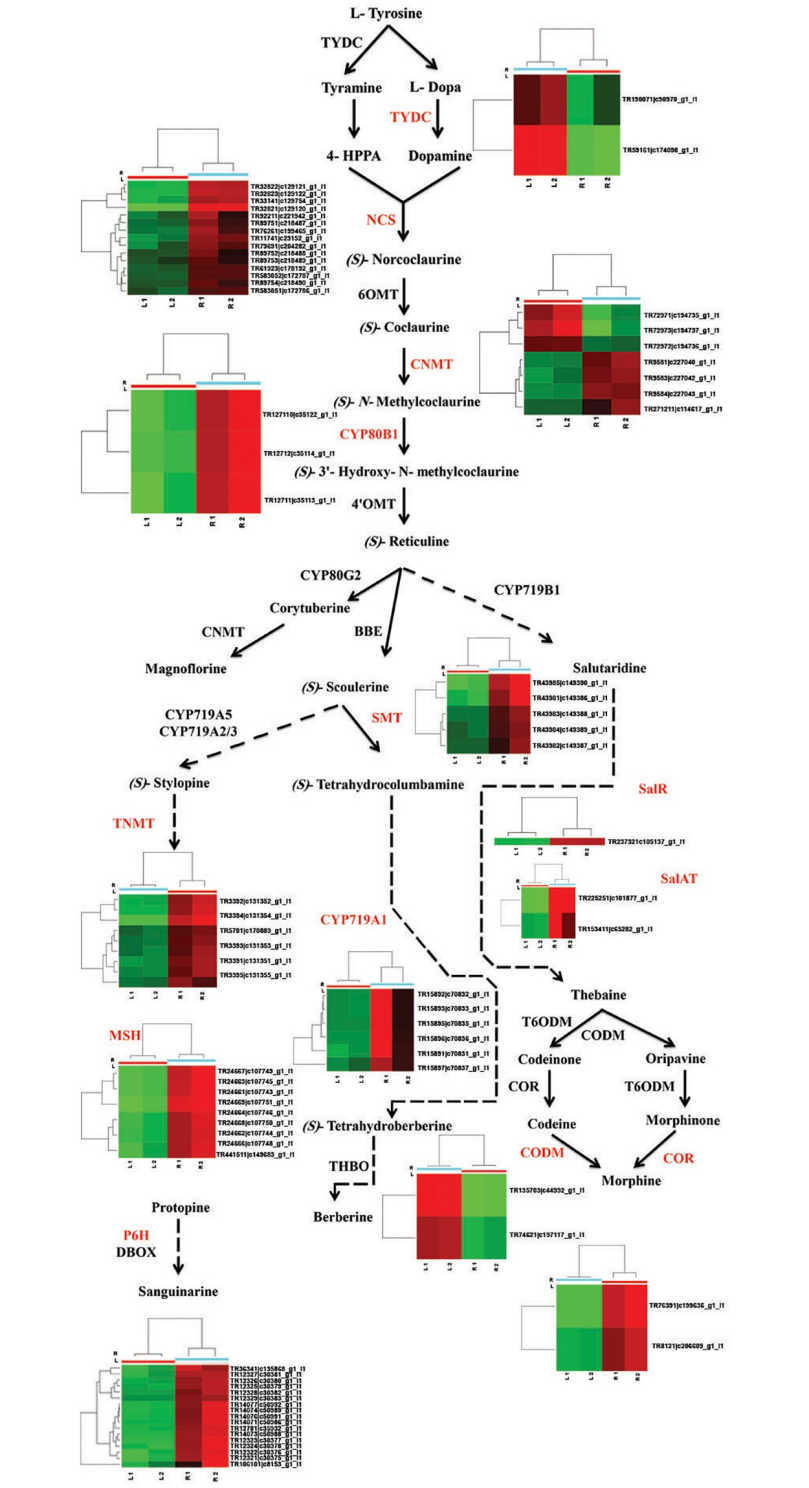
Schematic representation of the benzylisoquinoline alkaloid biosynthesis pathway and expression of the genes for pathway enzymes in *Chelidonium majus*.

The principal pathway for metabolism of morphinans (codeine and morphine), protoberberines (berberine) and benzophenanthridines (sanguinarine) starts with the formation of (S)-reticuline. Berberine and sanguinarine are found simultaneously in only a few species [52]. The biosynthesis of (S)-reticuline begins with the conversion of tyrosine to dopamine and 4-hydroxyphenylacetaldehyde (4HPAA) [53–54]. Tyrosine/dopa decarboxylase (*TYDC*), which yields tyramine or dopamine, has been isolated from a range of plant species [55–56]. *TYDC* gene was found to be strongly expressed in root (Fig 1), displaying different expression patterns in different organs. Previous studies have shown that *TYDC*s are regulated by multiple factors and are differentially expressed in response to elicitor treatments [57–59].

Norcoclaurine synthase (*NCS*) yields (S)-norcoclaurine through the condensation of 4-HPAA and dopamine. The *NCS* gene sequence was initially isolated from meadow rue (*Thalictrum flavum*) [54] and then from opium poppy [60]. In our study, the expression of *NCS* was significantly higher in root than in leaves, which is consistent with the results of previous studies, although *NCS*-specific mRNA has been detected in flower buds and germinating seeds [61–62]. Coclaurine N-methyltransferase (*CNMT*), which is expressed in roots, stems, flower buds, and at lower levels in leaves [63] is an N-methyltransferase which converts (S)-Coclaurine to (S)-N-methylcoclaurine [64]. In this study, gene-specific transcripts of *CNMT* were detected in both tissues. (S)-N-methylcoclaurine 3′-hydroxylase (CYP80B1) is a P450 hydroxylase [65]. Three transcripts related to CYP80B1 showed high levels of expression in rootManuscript, which is consistent with previously published results [66]. We detected three, two, and four genes in the sanguinarine, berberine, and morphine pathways, respectively. In the sanguinarine pathway, the gene for tetrahydroprotoberberine *cis*-N-methyltransferase (*TNMT*), which converts (S)-stylopine to (S)-*cis*-N-methylstylopine, showed higher expression levels in leave as compared to root, but the methylstylopine hydroxylase (*MSH*) and protopine 6-hydroxylase (*P6*H) genes had higher expression levels in root. MSH and P6H both belong to the P450 enzyme family [67]. Most previous studies showed that *TNMT*, *MSH*, and *P6H* are highly expressed in root, with the lowest expression levels detected in leave, fruits, or bulb initiation [68]. However, Liscombe and Facchini (2007) measured the highest levels of *TNMT* activity in the stem and leaf tissues of opium poppy, with lower levels in roots and flower buds, which is consistent with our results [69]. In the berberine pathway, genes for (S)-scoulerine-9-O-methyltransferase (SMT) [70] and (S)-canadine synthase (CYP719A1) [71] were both up-regulated in root, but of four genes detected in the morphine pathway, three, including those for salutaridine reductase (SalR), salutaridinol 7-O-acetyltransferase (SalAT), and codeine O-demethylase (CODM), were up-regulated in root while transcription of the codeinone reductase (COR) gene was up-regulated in leaf. COR appears twice in the codeine and morphine pathway; (1) it catalyzes the NADPH-dependent reduction of codeinone to codeine [72], and (2) it is involved in the conversion of morphinone to morphine [73].

### Functional annotation and GO classification

**Fig 2:**
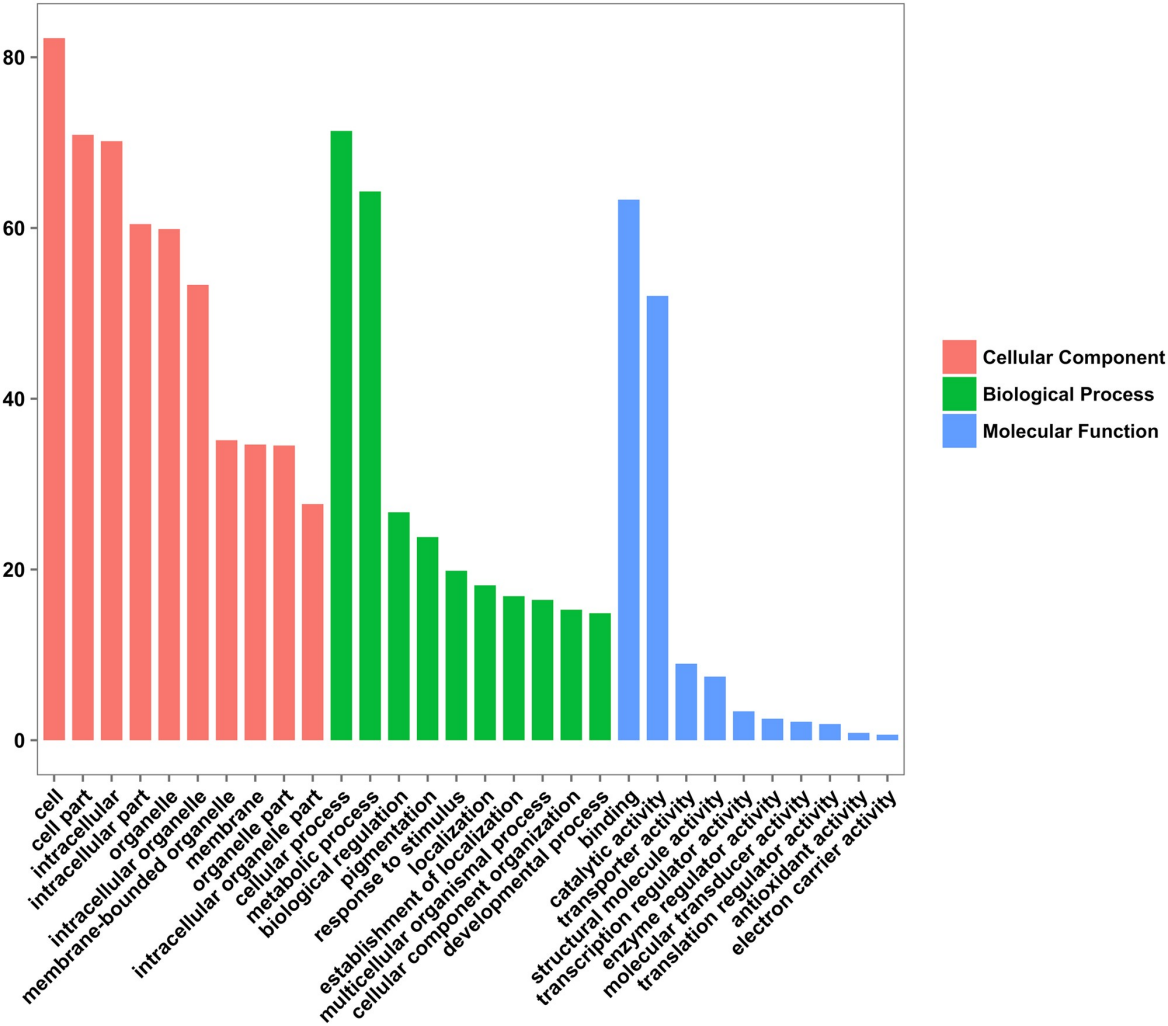
GO classification of genes expressed in *Chelidonium majus*. The bar chart shows the percentage of genes (Y-axis) belonging to the top 10 categories (X-axis) in the “cellular component”, “biological process”, and “molecular function” GO domains.

Gene annotation is one of the most important parts of transcriptome analysis, because it enables us to interpret the content of transcriptome assembly. A total of 97,275 (41.8%) and 196,640 sequences (84.5%) gave significant hits against the Swiss-Prot database using BLASTx and BLASTp searches, respectively. Furthermore, 14,894 unique Pfam protein motifs were assigned and 8,805 transcripts were predicted to encode proteins with signal peptides. Of the transcripts that returned BLASTx hits, 110,757 were associated with a total of 853,310 Gene Ontology (GO) terms. Of these annotated transcripts, 5,609 had only a single GO term. Fig 2 summarizes the percentage of genes belonging to the top 10 categories in the “biological process”, “cellular component”, and “molecular function” GO domains. Among the three main domains, “cellular component” was the most highly represented, and within this category most of the genes belonged to the “cell” class, followed by the “cell part” and “intracellular” classes. In the case of the “biological process” domain, the most abundant categories were “cellular process” and “metabolic process”, and for the “molecular function” domain, the predominant categories were “binding” and “catalytic activity”. The GO term abundance results are similar to those from a large number of transcriptome studies that have been reported for other non-model and medicinal plants, such as saffron [74], gardenia [75], safflower [76], and chrysanthemum [77]; however, compared to a previous study on *C*. *majus* [18], the distribution of genes in the three main ontologies was different. The most noticeable difference was observed in the distribution of genes in “molecular function” GO domain. Possible reasons for the discrepancies between our study and that of Nawrot et al. (2016) could include variations in the structure of the cDNA libraries and/or the number of sequences used to retrieve GO terms [18].

### Identification and analysis of transcription factor genes

**Fig 3:**
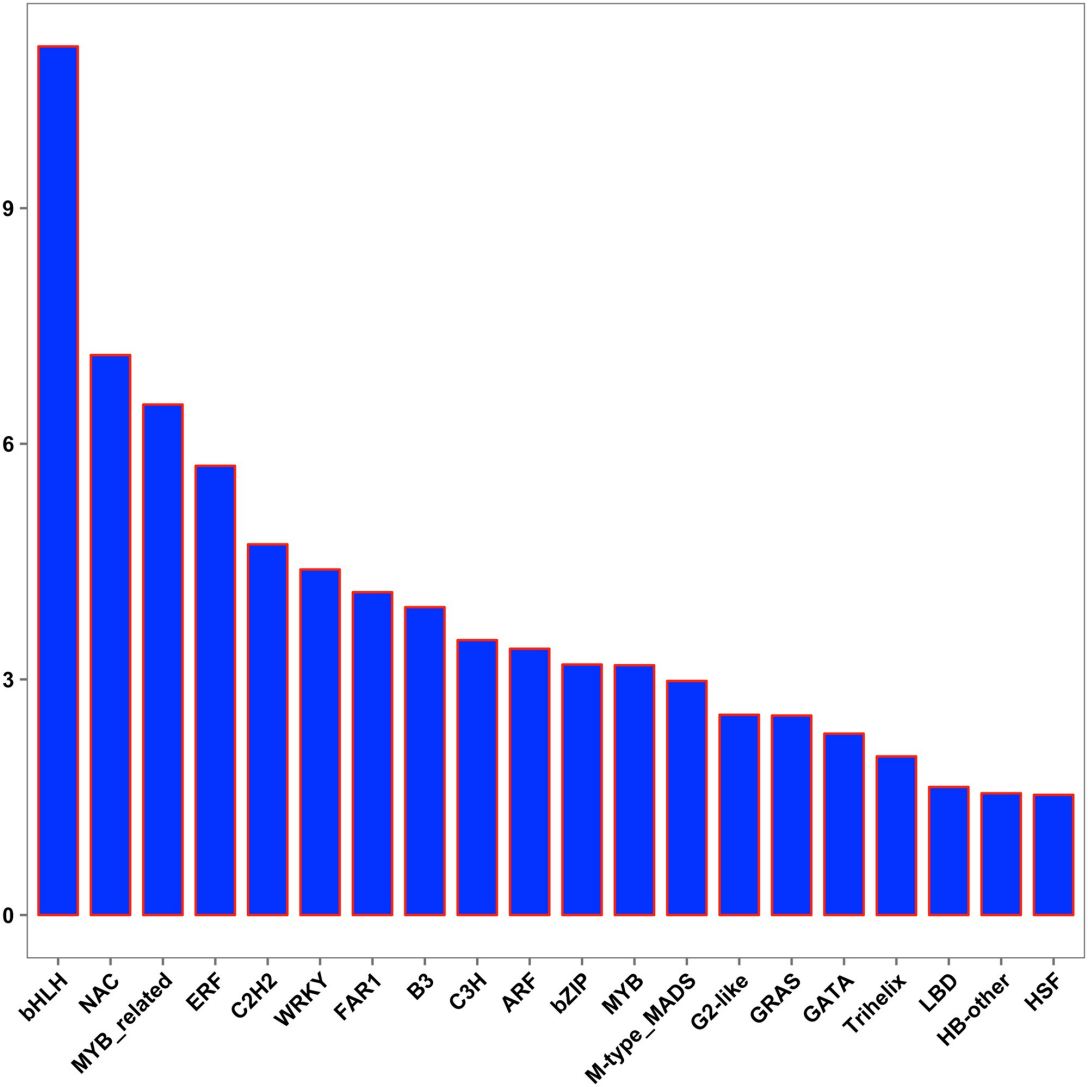
Percentages of *Chelidonium majus* transcripts (Y-axis) representing the top transcription factor families (X-axis) identified in this study.

Transcription factors (TFs) play multiple key roles in plants by controlling the synthesis of bioactive components, especially secondary metabolism and regulation of gene expression through DNA-binding and cis-acting elements [78–79]. Here, a total of 69,971 putative TF encoding transcripts were identified and further classified into 64 different families in the *C*. *majus* transcriptome. Among the transcription factor families, bHLH was the most highly represented, with 7,736 transcripts (11.06%), followed by NAC (4,992; 7.13%), MYB-related (4,545; 6.50%), ERF (4,003; 5.72%), C2H2 (3,300; 4.72%), and WRKY (3,077; 4.40%) (Fig 3).

Previous studies have demonstrated that the bHLH TFs could play major roles not only in the developmental processes including control of cell proliferation [80] and formation of trichome and light signal transduction [81], but also in the regulation of the expression of many genes which participate in the biosynthesis of plant secondary metabolites such as flavonoids and alkaloids [82]. In addition to bHLH, other TF families such as WRKY, MYB, and C2H2 are involved in secondary metabolism pathways. Two transcription factors, CjWRKY1, a WRKY-type TF [83] and CjbHLH1, a basic helix-loop-helix TF [84] have been identified in the alkaloid pathway to independently regulate berberine biosynthesis. CjWRKY1 is the first transcription factor which has been characterized to play a positive role in berberine synthesis in *Coptis japonica* [83]. CjbHLH1 is a non-MYC2-type bHLH TF, and two homologs, EcbHLH1-1 and EcbHLH1-2, that are associated with the regulation of sanguinarine synthesis, have been identified in the California poppy, *Eschscholiza californica* [85]. The ERF subfamily, which belongs to the AP2/ERF family, have only a single AP2/ERF domain, and are known to be involved in dehydration or ethylene responses [86]. ERF189 and ERF221/ORC1 in *N*. *tabacum* and ORCA2 and ORCA3 in *C*. *roseus* are members of the AP2/ERF TF family that have been identified as being involved in alkaloid biosynthesis [87]. MYB transcription factors control diverse biological processes such as the regulation of primary/secondary metabolism and hormone syntheses [88–90], whereas NAC family members participate in regulating plant growth and developmental processes [91–93].

### EST-SSR frequency and distribution

**Fig 4:**
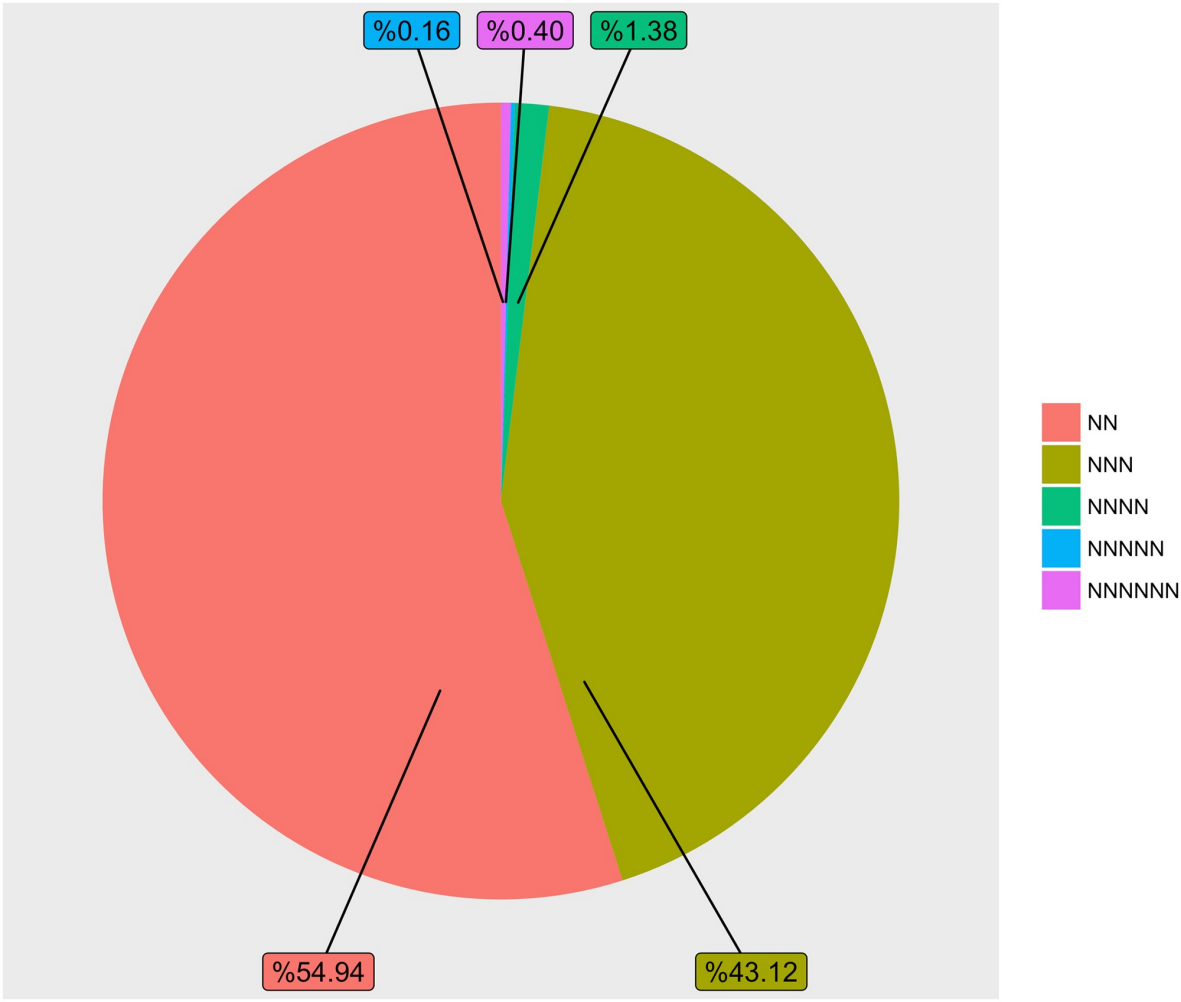
Expressed sequence tag simple sequence repeatss (SSRs) identified in the *Chelidonium majus* transcriptome. Distribution of SSRs in different length classes.

EST-SSRs have been extensively used in the study of genetic variation, evolutionary relationships, linkage mapping, and genotyping due to their abundance, high polymorphic information content, good reproducibility, and relative ease of use. At present, there are no studies addressing the genetic diversity and classification of *C*. *majus* germplasm resources based on EST-SSR markers, because have not been identified so far. In this study, for the first time, large-scale transcriptome sequencing was used to identify expressed sequence tag simple sequence repeats (EST-SSR) markers. To develop new markers for *C*. *majus*, all of the 232,701 transcripts generated by BinPacker were screened to find potential microsatellite motifs using the MISA search tool. Due to both sequencing and assembly errors, mononucleotide repeats may not be reliable, so we excluded them from further analyses. A total of 39,841 EST-SSRs (2–6 nt) were identified in 45,277 (19.45%) transcripts (Fig 4), and 15,293 sequences were found to contain more than one EST-SSR motif. The dinucleotide repeat motifs were the most abundant (21,887 or 54.94%), followed by trinucleotide repeats (17,180 or 43.12%), and only 540 (1.38%), 64 (0.16%), and 160 (0.40%) of the identified EST-SSRs harbored predominately tetra-, penta-, and hexanucleotide repeat motifs respectively. Within EST-SSR data sets, dinucleotide repeat frequencies are usually higher than trinucleotide repeat frequencies. This is supported by studies on medicinal plants such as *Andrographis paniculata* [94], *Ginkgo biloba* L. [95], *Gleditsia sinensis* [96], *Crocus sativus* [74], *Boea clarkeana* [97], *Phyllanthus amarus* [98], and *Cinnamomum longepaniculatum* [99]. However, the tri-nucleotide repeats are more frequent than di-nucleotide repeats in some other medicinal plants such as *Mucuna pruriens* [100] and *Epimedium sagittatum* [101]. These distribution frequencies vary with respect to the different plant species, the employed datasets, and the tools and standards used for EST-SSR searches and identification.

**Fig 5:**
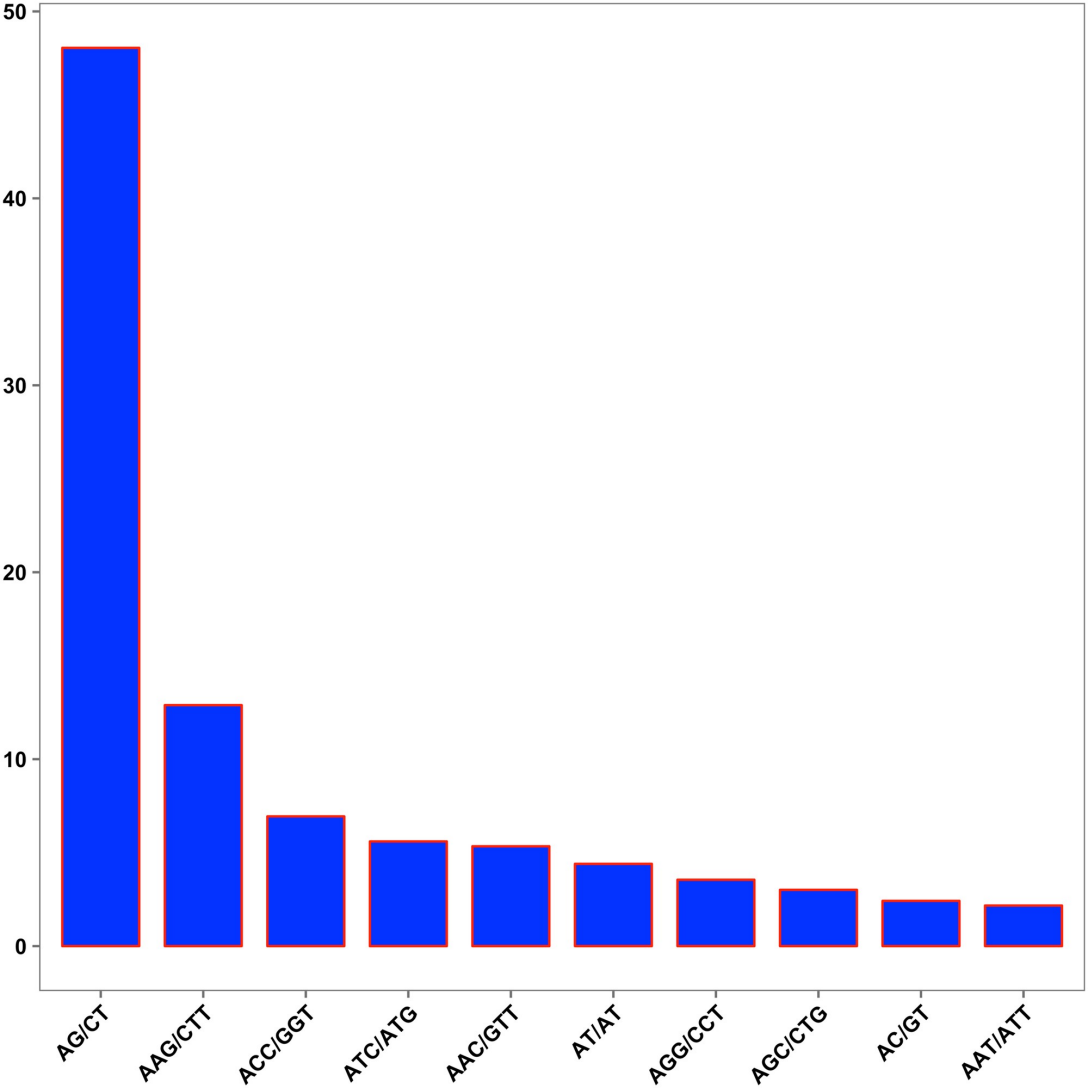
Expressed sequence tag simple sequence repeatss (SSRs) identified in the *Chelidonium majus* transcriptome. The y-axis indicates frequencies of the 10 most abundant SSRs motifs. The x-axis indicates 10 groups of SSRs motifs.

Among the dinucleotide repeat motifs, we found that AG/CT was the most common (48.05%) in *C*. *majus*, and this is the case for plants in general [102]. The presence of CT repeat sequences in 5′-UTRs is probably related to reverse transcription and has a significant role in gene regulation. Of the trinucleotide repeats, AAG/CTT was the most frequent motif (12.89%) in *C*. *majus*, followed by ACC/GGT (6.94%) (Fig 5). The (AAG/CTT)n repeats and their complements are the most common tri-nucleotide repeat motifs in plants [103]. We succeeded in identifying several novel EST-SSRs which were linked to unigenes that putatively encode enzymes involved in morphine and sanguinarine biosynthesis. Finally we designed high-quality primers to amplify these potential EST-SSR loci (Table 2). Our findings will enrich the molecular marker resources and help spearhead molecular genetic research on *C*. *majus*.

**Table 2:** Identification of SSR motifs in putative morphine and sanguinarine biosynthesis genes.

ID	SSR	Forward Primer (5'-3')	Tm(°C)	size	Reverse Primer (5'-3')	Tm(°C)	size	PS (bp)**
S-norcoclaurine(NCS)	(AAG)5	AAGAAACCTGCAGCAGAGGA	60.134	20	TCTTGTAGGTCTCGACGTTCTTC	59.936	23	157
S-norcoclaurine(NCS)	(TA)8	CCTGAGGTGGGTGTCAAGAT	59.962	20	CGTGGTAGTAGAAGATCCAATTAAA	57.965	25	214
Salutaridinol 7-O-acetyltransferase(SalAT)	(TCA)5	CATCAGTGTCGGTGTTGTCC	60.005	20	TGGAGGAATTGGTGGGTAAA	60.162	20	189
Salutaridinol 7-O-acetyltransferase(SalAT)	(ACA)5	GCAGTTGCGCTTGAATATGA	59.983	20	GAAGACGACGATGATGACCA	59.637	20	227
(S)-tetrahydroprotoberberine(TNMT)	(CT)8	CTTCCTCCCATCACCCACTA	59.92	20	CCTCTCCCATTGATGCCTAA	60.029	20	256
(S)-tetrahydroprotoberberine(TNMT)	(GAC)5	CGACGGAGGATGAGTTGATT	60.073	20	CCAGACGTTGTAGTCCGGTT	60.028	20	238
(S)-tetrahydroprotoberberine(TNMT)	(GGT)5	CCGGACTACAACGTCTGGAT	59.989	20	GGTTTTCTTTCTGCCGATGA	60.192	20	118
(S)-tetrahydroprotoberberine(TNMT)	(AT)7	CACAATTAGGCCCACATCAA	59.395	20	GCCTTGCATGAATATGCTGA	59.799	20	203
Methyltetrahydroprotoberberine 14-monooxygenase (MSH)	(AT)6	ACACCAACCAAAGCAAAAGC	60.154	20	GGAGGTGCAAAGGTTGACAT	59.973	20	219
NADPH-dependent codeinone reductase(COR)	(AG)8	TTCCCATTAGGCAACAATCC	59.762	20	TTGGCATCTCCCTACCTGAG	60.21	20	226

** PS: PRODUCT1 size,

### Discovery of miRNAs

A high stringency filtering approach on BLAST results identified a total of 104 potential miRNAs belonging to 108 sequences that were retained for secondary structure analysis. After filtering based on secondary structure, nine folded miRNA precursors were predicted from nine different families for the first time in *C*. *majus* (Table 3). In this study, the identified precursors had high MFEI values (0.71–0.83) with an average of 0.76, which is higher than that of rRNAs (0.59), tRNAs (0.64) or mRNAs (0.62–0.66) [40].

**Table 3:** High-probability miRNAs proposed for *Chelidonium majus*.

miRNA family	miRNA ID	Mature sequence	Mismatch	ΔG = -kcal/mol	MFEI
mir477	mes-miR477h	ACUCUCCCUCAAGGGCUUCAG	4	82.6	0.77
mir319	ath-miR319a	UUGGACUGAAGGGAGCUCCCU	2	116.2	0.83
mir396	cca-miR396c	UUCAAGAAAGCUGUGGGAAAA	1	117.3	0.77
mir159	pde-miR159	UUUGGUUUGAAGGGAGCUCUA	4	116.5	0.74
mir828	vvi-miR828a	UCUUGCUCAAAUGAGUAUUCCA	3	102	0.71
mir171	ctr-miR171	UUGAGCCGCGUCAAUAUCUCC	1	125.80	0.73
mir167	ath-miR167d	UGAAGCUGCCAGCAUGAUCUGG	2	105.60	0.71
mir4376	sly-miR4376	ACGCAGGAGAGAUGAUGCUGGA	5	109.6	0.81
mir169	gma-miR169d	UGAGCCAAGGAUGACUUGCCGGU	4	98.1	0.77

MFEI: Minimal folding free energy index.

Most mature miRNAs are evolutionarily conserved between species within the plant kingdom, some of which have a large number of potential targets. Of these, miR319 regulates transcription factors belonging to the TCP family which regulate plant developmental processes such as leaf morphogenesis in *Arabidopsis* [104]. miR396 is necessary for normal development in *Arabidopsis*, and regulates the Growth-Regulating Factor (GRF) family of transcription factors. GRFs are known to control cell proliferation in *Arabidopsis* leaves [105]. miR159 has a very similar sequence to miR319 but regulates different genes [106]. miR828 appears to target transcription factor genes for DNA binding domain-containing proteins such as CONSTANS-like 5 related cluster protein and zinc finger protein-B box [107]. Most studies have shown that the miR171 family negatively regulates (decreases) primary root elongation and shoot branching by targeting GRAS gene family members [108]. Auxin Response Factors (ARFs), proteins that play important roles in plant growth and development, have been reported to be targets of the miR167 family in *Oryza sativa* [109]. miR169 is mostly expressed in the roots and regulates CCAAT motif-binding transcription factors [107].

### Construction of orthogroups across multiple species of Papaveracea

**Fig 6:**
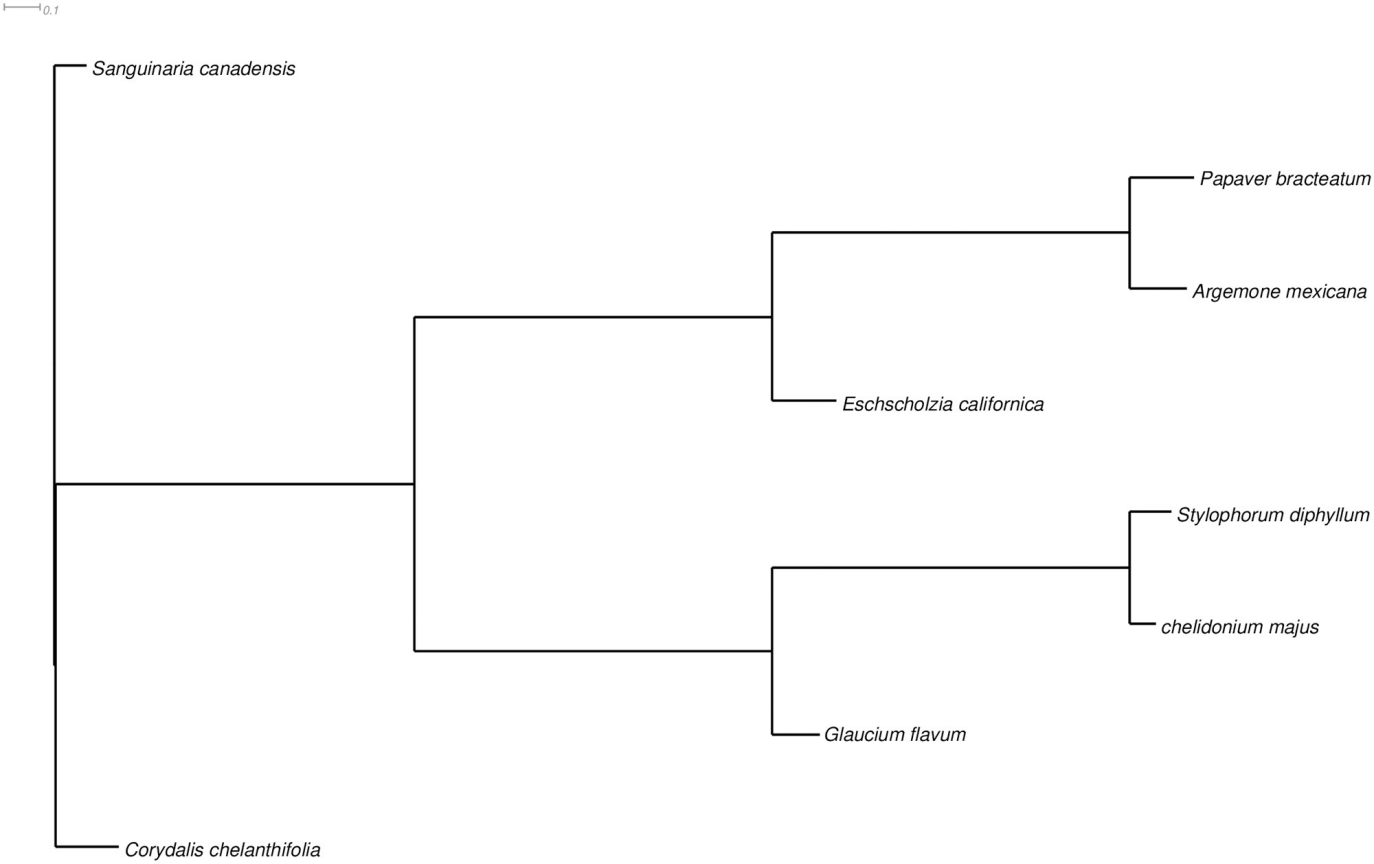
Phylogenetic tree showing eight BIA-accumulating plant species from the concatenated orthogroups using OrthoFinder. The tree is drawn to scale, with branch lengths in the same units as those of the evolutionary distances used to infer the phylogenetic tree.

To facilitate comparative studies and to demonstrate the utility of transcriptome assemblies for phylogenetic analysis, candidate coding regions generated by TransDecoder from transcriptome assemblies of seven species were compared with potential proteins based on ORF predictions in the *C*. *majus* transcriptome using OrthoFinder. The number of shared orthogroups between each pair of species ranged from 10,925 (between *Eschscholzia californica* and *Papaver bracteatum*) to 15,498 (between *C*. *majus* and *Argemone mexicana*). A total of 8,483 orthogroups were identified among all species present, and there were 59 single-gene orthogroups in our species comparison. The family Papaveraceae is divided into four subfamilies based on critical details of the morphological traits [110]. In this study, with the exception of *Corydalis cheilanthifolia*, which belongs to the Fumarioideae subfamily, all other species belong to the Papaveroideae subfamily. The species tree strongly supports genetic relationship between *C*. *majus* and *S*. *diphyllum* (Fig 6). This tree suggests that *C*. *majus* and *S*. *diphyllum* are the most divergent from *C*. *cheilanthifolia* in the Fumarioideae subfamily.

## Conclusions

In the current study, we generated and characterized a fully annotated and deep-sequencing transcriptome assembly for leaves and root tissues of *C*. *majus*. This represents an important initial resource that will enable further studies on the molecular mechanisms of bioactive alkaloids biosynthesis, as well as for studies of the molecular genetics and functional genomics of this important medicinal plant. Based on transcriptome assembly metrics, BinPacker was found to be the best among all the assemblers used in this study. Generally, our analysis revealed that most of the genes involved in the sanguinarine, berberine, and morphine pathways are broadly expressed in root. We observed that relatively few of these genes are up-regulated in leaves. Our results also showed that the most frequent transcription factor families represented here are involved in regulating secondary metabolism pathways, especially those for alkaloid biosynthesis. Development of a large number of EST-SSR markers and the design of high-quality PCR primers for potential EST-SSR loci amplification in the *C*. *majus* transcriptome will be useful for evaluating genetic diversity and also in marker-assisted breeding in *C*. *majus*. Furthermore, our computational methods enabled the identification of a set of potential miRNAs which were previously unknown for this plant.

## Abbreviations

BIAs: benzylisoquinoline alkaloids
DGE: differential gene expression
EST-SSRs: expressed sequence tag simple sequence repeats
GO: gene ontology
RMBT: reads mapped back to the transcriptome assembly
TFs: Transcription factors
